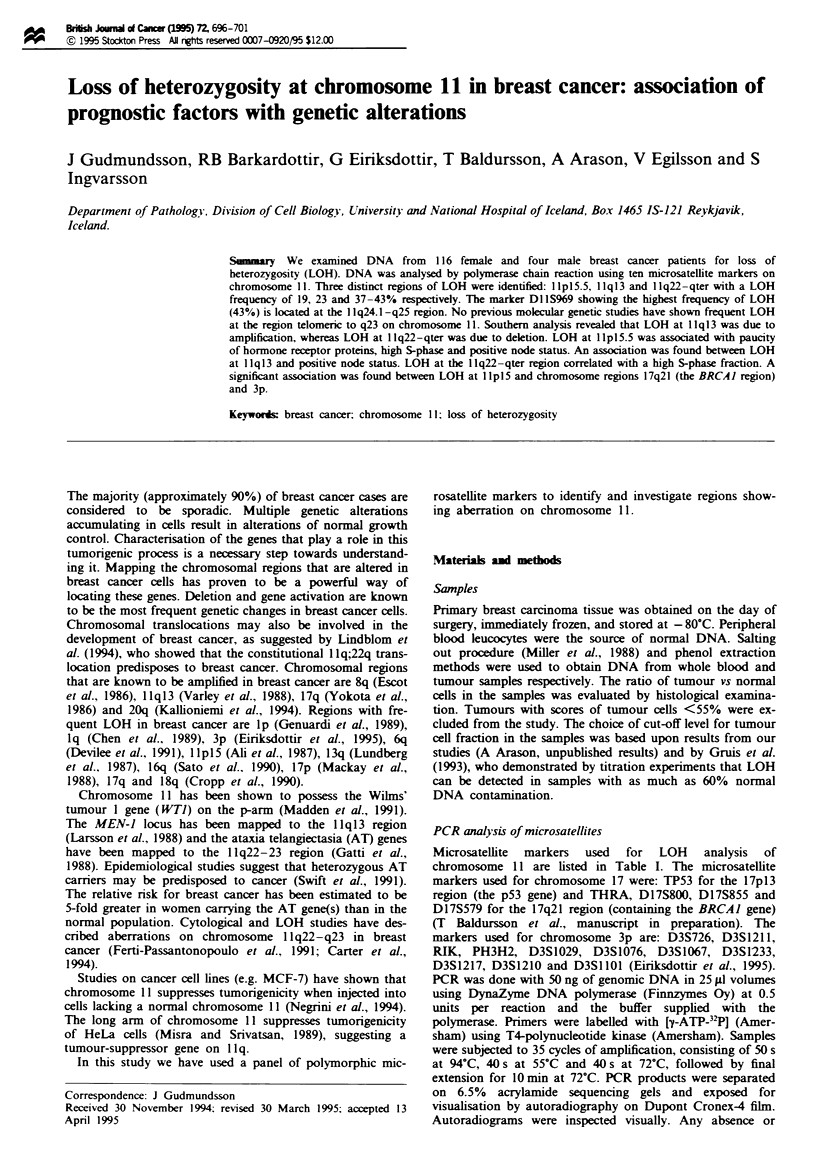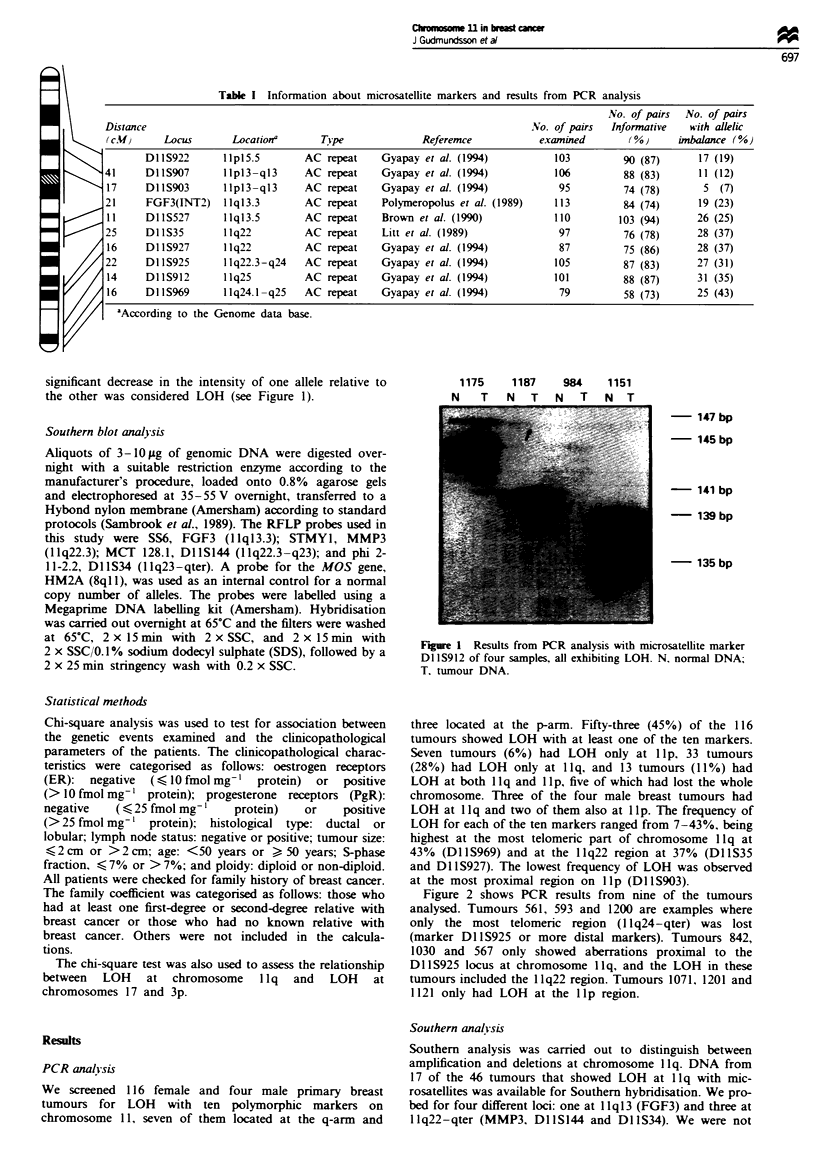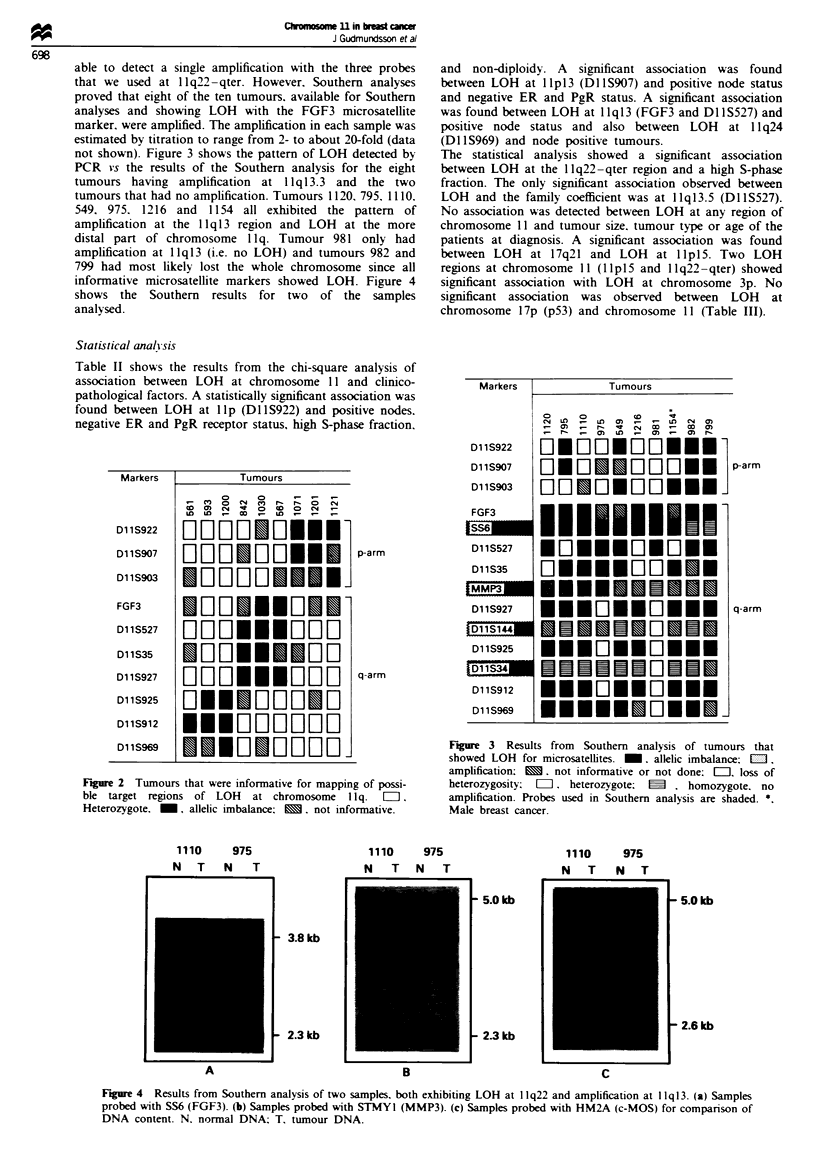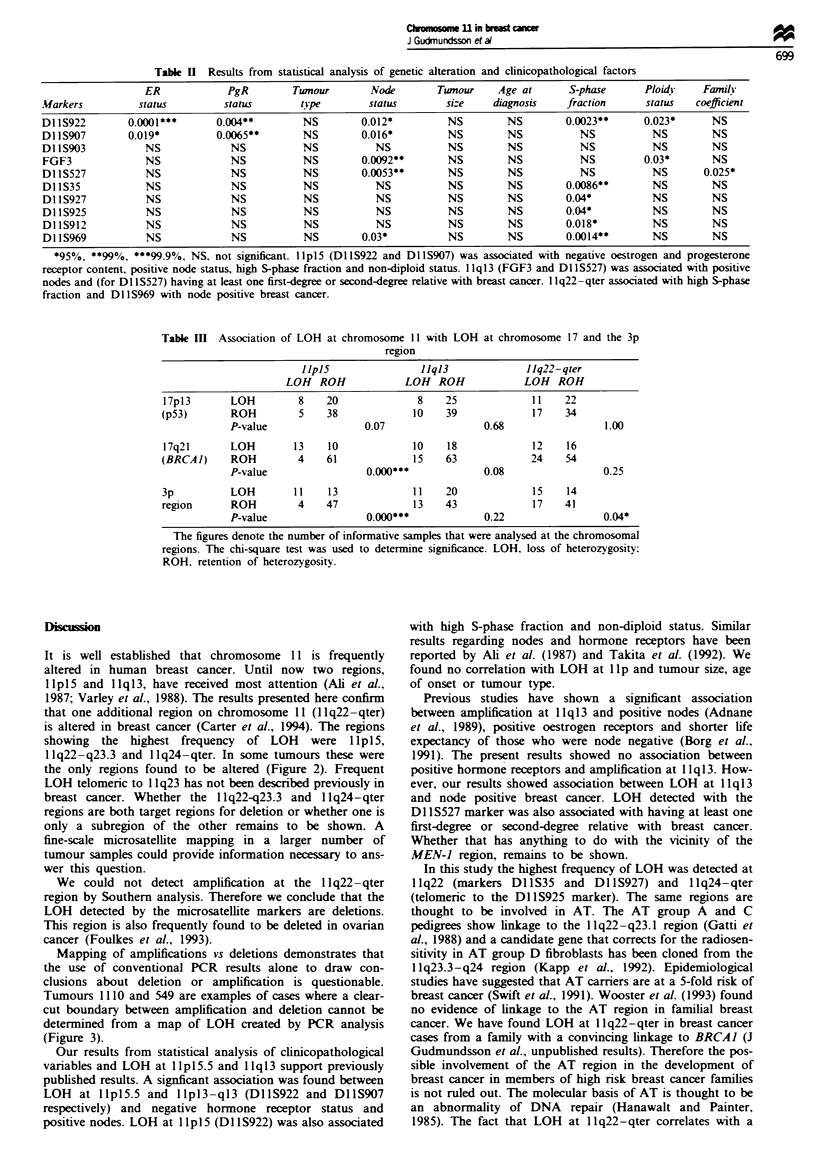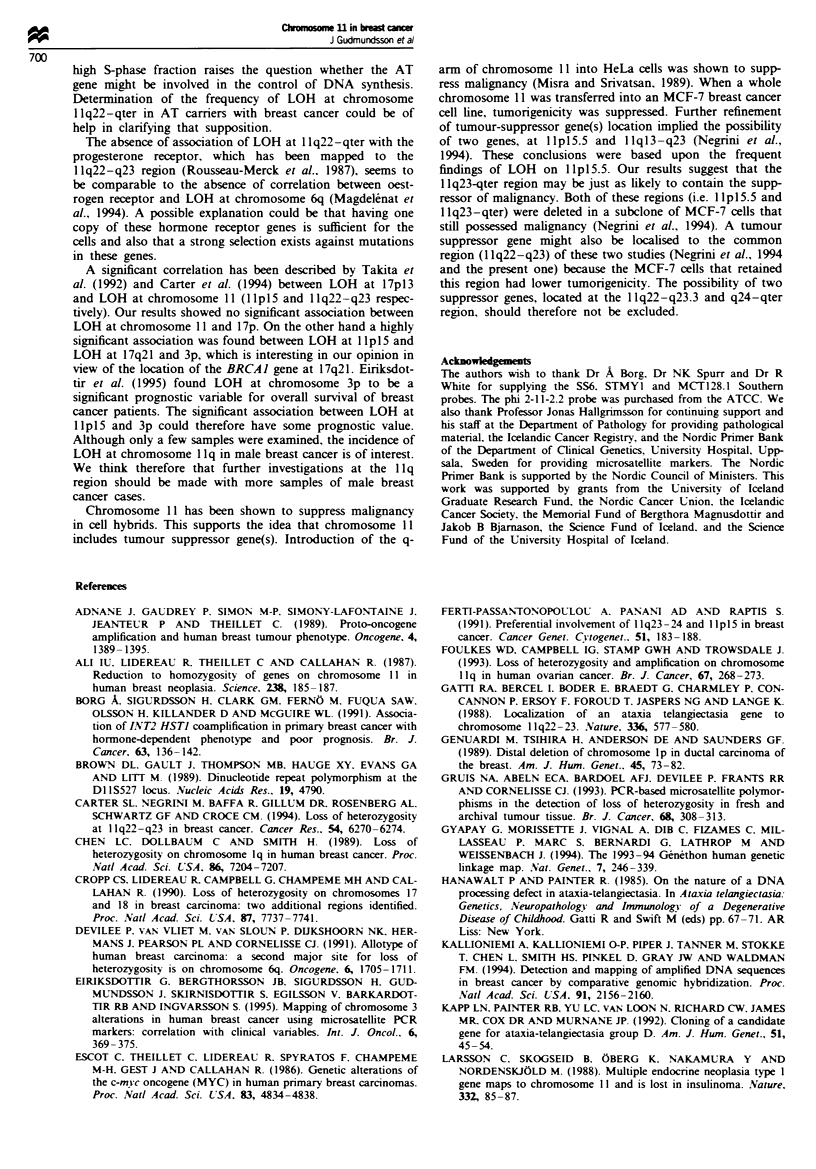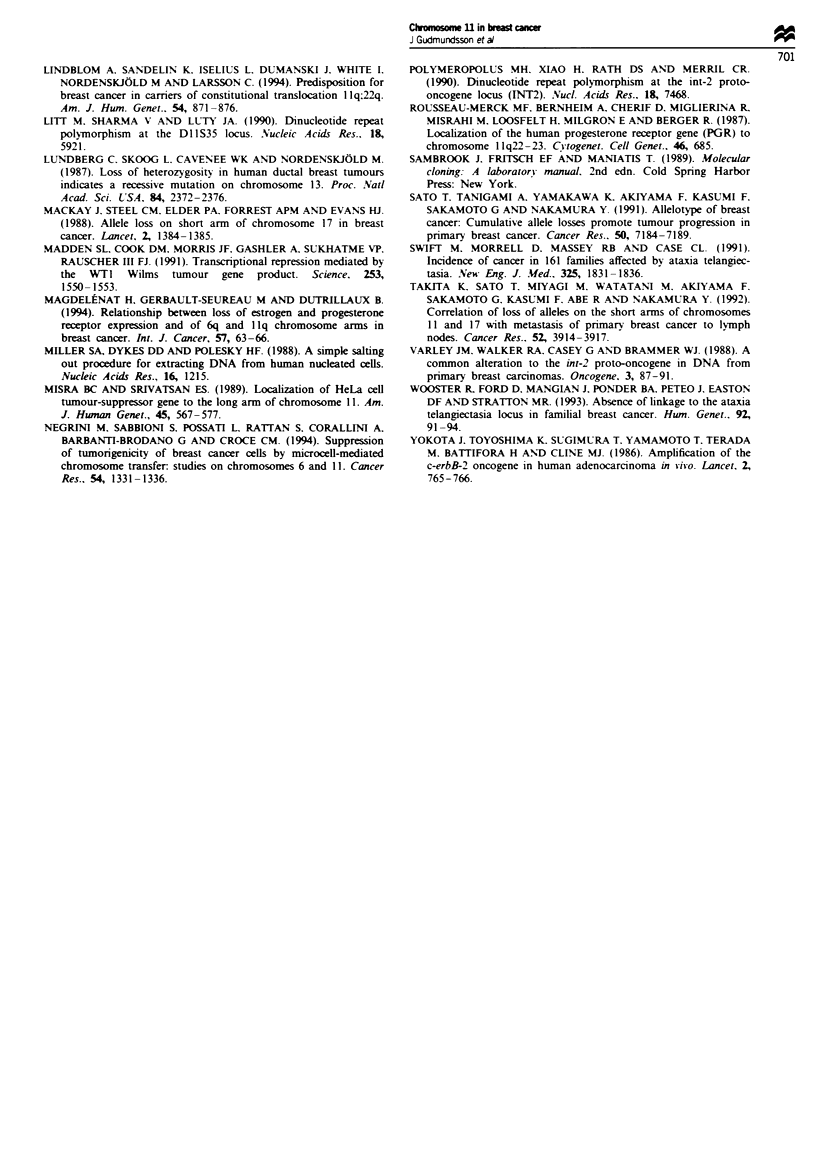# Loss of heterozygosity at chromosome 11 in breast cancer: association of prognostic factors with genetic alterations.

**DOI:** 10.1038/bjc.1995.396

**Published:** 1995-09

**Authors:** J. Gudmundsson, R. B. Barkardottir, G. Eiriksdottir, T. Baldursson, A. Arason, V. Egilsson, S. Ingvarsson

**Affiliations:** Department of Pathology, University and National Hospital of Iceland, Reykjavik.

## Abstract

**Images:**


British Journal of Cancer (1995) 72, 696–701

© 1995 Stockton Press All rights reserved 0007–0920/95 $12.00

### Loss of heterozygosity at chromosome 11 in breast cancer: association of prognostic factors with genetic alterations

J Gudmundsson, RB Barkardottir, G Eiriksdottir, T Baldursson, A Arason, V Egilsson and S Ingvarsson

*Department of Pathology, Division of Cell Biology, University and National Hospital of Iceland, Box 1465 IS-121 Reykjavik, Iceland.*

**Summary** We examined DNA from 116 female and four male breast cancer patients for loss of heterozygosity (LOH). DNA was analysed by polymerase chain reaction using ten microsatellite markers on chromosome 11. Three distinct regions of LOH were identified: 11p15.5, 11q13 and 11q22–qter with a LOH frequency of 19, 23 and 37–43% respectively. The marker D11S969 showing the highest frequency of LOH (43%) is located at the 11q24.1–q25 region. No previous molecular genetic studies have shown frequent LOH at the region telomeric to q23 on chromosome 11. Southern analysis revealed that LOH at 11q13 was due to amplification, whereas LOH at 11q22–qter was due to deletion. LOH at 11p15.5 was associated with paucity of hormone receptor proteins, high S-phase and positive node status. An association was found between LOH at 11q13 and positive node status. LOH at the 11q22–qter region correlated with a high S-phase fraction. A significant association was found between LOH at 11p15 and chromosome regions 17q21 (the *BRCA1* region) and 3p.

**Keywords:** breast cancer; chromosome 11; loss of heterozygosity

The majority (approximately 90%) of breast cancer cases are considered to be sporadic. Multiple genetic alterations accumulating in cells result in alterations of normal growth control. Characterisation of the genes that play a role in this tumorigenic process is a necessary step towards understanding it. Mapping the chromosomal regions that are altered in breast cancer cells has proven to be a powerful way of locating these genes. Deletion and gene activation are known to be the most frequent genetic changes in breast cancer cells. Chromosomal translocations may also be involved in the development of breast cancer, as suggested by Lindblom *et al.* (1994), who showed that the constitutional 11q;22q translocation predisposes to breast cancer. Chromosomal regions that are known to be amplified in breast cancer are 8q (Escot *et al.*, 1986), 11q13 (Varley *et al.*, 1988), 17q (Yokota *et al.*, 1986) and 20q (Kallioniemi *et al.*, 1994). Regions with frequent LOH in breast cancer are 1p (Genuardi *et al.*, 1989), 1q (Chen *et al.*, 1989), 3p (Eiriksdottir *et al.*, 1995), 6q (Devilee *et al.*, 1991), 11p15 (Ali *et al.*, 1987), 13q (Lundberg *et al.*, 1987), 16q (Sato *et al.*, 1990), 17p (Mackay *et al.*, 1988), 17q and 18q (Cropp *et al.*, 1990).

Chromosome 11 has been shown to possess the Wilms' tumour 1 gene (*WT1*) on the p-arm (Madden *et al.*, 1991). The *MEN-1* locus has been mapped to the 11q13 region (Larsson *et al.*, 1988) and the ataxia telangiectasia (AT) genes have been mapped to the 11q22–23 region (Gatti *et al.*, 1988). Epidemiological studies suggest that heterozygous AT carriers may be predisposed to cancer (Swift *et al.*, 1991). The relative risk for breast cancer has been estimated to be 5-fold greater in women carrying the AT gene(s) than in the normal population. Cytological and LOH studies have described aberrations on chromosome 11q22–q23 in breast cancer (Ferti-Passantonopoulo *et al.*, 1991; Carter *et al.*, 1994).

Studies on cancer cell lines (e.g. MCF-7) have shown that chromosome 11 suppresses tumorigenicity when injected into cells lacking a normal chromosome 11 (Negrini *et al.*, 1994). The long arm of chromosome 11 suppresses tumorigenicity of HeLa cells (Misra and Srivatsan, 1989), suggesting a tumour-suppressor gene on 11q.

In this study we have used a panel of polymorphic microsatellite markers to identify and investigate regions showing aberration on chromosome 11.

Correspondence: J Gudmundsson
Received 30 November 1994; revised 30 March 1995; accepted 13 April 1995

### Materials and methods

### *Samples*

Primary breast carcinoma tissue was obtained on the day of surgery, immediately frozen, and stored at −80°C. Peripheral blood leucocytes were the source of normal DNA. Salting out procedure (Miller *et al.*, 1988) and phenol extraction methods were used to obtain DNA from whole blood and tumour samples respectively. The ratio of tumour *vs* normal cells in the samples was evaluated by histological examination. Tumours with scores of tumour cells <55% were excluded from the study. The choice of cut-off level for tumour cell fraction in the samples was based upon results from our studies (A Arason, unpublished results) and by Gruis *et al.* (1993), who demonstrated by titration experiments that LOH can be detected in samples with as much as 60% normal DNA contamination.

### *PCR analysis of microsatellites*

Microsatellite markers used for LOH analysis of chromosome 11 are listed in Table I. The microsatellite markers used for chromosome 17 were: TP53 for the 17p13 region (the p53 gene) and THRA, D17S800, D17S855 and D17S579 for the 17q21 region (containing the *BRCA1* gene) (T Baldursson *et al.*, manuscript in preparation). The markers used for chromosome 3p are: D3S726, D3S1211, RIK, PH3H2, D3S1029, D3S1076, D3S1067, D3S1233, D3S1217, D3S1210 and D3S1101 (Eiriksdottir *et al.*, 1995). PCR was done with 50 ng of genomic DNA in 25 µl volumes using DynaZyme DNA polymerase (Finnzymes Oy) at 0.5 units per reaction and the buffer supplied with the polymerase. Primers were labelled with [γ-ATP-$^{32}$P] (Amersham) using T4-polynucleotide kinase (Amersham). Samples were subjected to 35 cycles of amplification, consisting of 50 s at 94°C, 40 s at 55°C and 40 s at 72°C, followed by final extension for 10 min at 72°C. PCR products were separated on 6.5% acrylamide sequencing gels and exposed for visualisation by autoradiography on Dupont Cronex-4 film. Autoradiograms were inspected visually. Any absence or

**Table I** Information about microsatellite markers and results from PCR analysis

| Distance (cM) | Locus | Location[a] | Type | Referemce | No. of pairs examined | No. of pairs Informative (%) | No. of pairs with allelic imbalance (%) |
|---|---|---|---|---|---|---|---|
| | D11S922 | 11p15.5 | AC repeat | Gyapay *et al.* (1994) | 103 | 90 (87) | 17 (19) |
| 41 | D11S907 | 11p13-q13 | AC repeat | Gyapay *et al.* (1994) | 106 | 88 (83) | 11 (12) |
| 17 | D11S903 | 11p13-q13 | AC repeat | Gyapay *et al.* (1994) | 95 | 74 (78) | 5 (7) |
| 21 | FGF3(INT2) | 11q13.3 | AC repeat | Polymeropolus *et al.* (1989) | 113 | 84 (74) | 19 (23) |
| 11 | D11S527 | 11q13.5 | AC repeat | Brown *et al.* (1990) | 110 | 103 (94) | 26 (25) |
| 25 | D11S35 | 11q22 | AC repeat | Litt *et al.* (1989) | 97 | 76 (78) | 28 (37) |
| 16 | D11S927 | 11q22 | AC repeat | Gyapay *et al.* (1994) | 87 | 75 (86) | 28 (37) |
| 22 | D11S925 | 11q22.3-q24 | AC repeat | Gyapay *et al.* (1994) | 105 | 87 (83) | 27 (31) |
| 14 | D11S912 | 11q25 | AC repeat | Gyapay *et al.* (1994) | 101 | 88 (87) | 31 (35) |
| 16 | D11S969 | 11q24.1-q25 | AC repeat | Gyapay *et al.* (1994) | 79 | 58 (73) | 25 (43) |

[a]According to the Genome data base.

significant decrease in the intensity of one allele relative to the other was considered LOH (see Figure 1).

*Southern blot analysis*

Aliquots of 3-10 μg of genomic DNA were digested overnight with a suitable restriction enzyme according to the manufacturer's procedure, loaded onto 0.8% agarose gels and electrophoresed at 35-55 V overnight, transferred to a Hybond nylon membrane (Amersham) according to standard protocols (Sambrook *et al.*, 1989). The RFLP probes used in this study were SS6, FGF3 (11q13.3); STMY1, MMP3 (11q22.3); MCT 128.1, D11S144 (11q22.3-q23); and phi 2-11-2.2, D11S34 (11q23-qter). A probe for the *MOS* gene, HM2A (8q11), was used as an internal control for a normal copy number of alleles. The probes were labelled using a Megaprime DNA labelling kit (Amersham). Hybridisation was carried out overnight at 65°C and the filters were washed at 65°C, 2 × 15 min with 2 × SSC, and 2 × 15 min with 2 × SSC/0.1% sodium dodecyl sulphate (SDS), followed by a 2 × 25 min stringency wash with 0.2 × SSC.

*Statistical methods*

Chi-square analysis was used to test for association between the genetic events examined and the clinicopathological parameters of the patients. The clinicopathological characteristics were categorised as follows: oestrogen receptors (ER): negative ($\leqslant 10$ fmol mg$^{-1}$ protein) or positive ($>10$ fmol mg$^{-1}$ protein); progesterone receptors (PgR): negative ($\leqslant 25$ fmol mg$^{-1}$ protein) or positive ($>25$ fmol mg$^{-1}$ protein); histological type: ductal or lobular; lymph node status: negative or positive; tumour size: $\leqslant 2$ cm or $>2$ cm; age: $<50$ years or $\geqslant 50$ years; S-phase fraction, $\leqslant 7$% or $>7$%; and ploidy: diploid or non-diploid. All patients were checked for family history of breast cancer. The family coefficient was categorised as follows: those who had at least one first-degree or second-degree relative with breast cancer or those who had no known relative with breast cancer. Others were not included in the calculations.

The chi-square test was also used to assess the relationship between LOH at chromosome 11q and LOH at chromosomes 17 and 3p.

### Results

*PCR analysis*

We screened 116 female and four male primary breast tumours for LOH with ten polymorphic markers on chromosome 11, seven of them located at the q-arm and three located at the p-arm. Fifty-three (45%) of the 116 tumours showed LOH with at least one of the ten markers. Seven tumours (6%) had LOH only at 11p, 33 tumours (28%) had LOH only at 11q, and 13 tumours (11%) had LOH at both 11q and 11p, five of which had lost the whole chromosome. Three of the four male breast tumours had LOH at 11q and two of them also at 11p. The frequency of LOH for each of the ten markers ranged from 7-43%, being highest at the most telomeric part of chromosome 11q at 43% (D11S969) and at the 11q22 region at 37% (D11S35 and D11S927). The lowest frequency of LOH was observed at the most proximal region on 11p (D11S903).

**Figure 1** Results from PCR analysis with microsatellite marker D11S912 of four samples, all exhibiting LOH. N, normal DNA; T, tumour DNA.

Figure 2 shows PCR results from nine of the tumours analysed. Tumours 561, 593 and 1200 are examples where only the most telomeric region (11q24-qter) was lost (marker D11S925 or more distal markers). Tumours 842, 1030 and 567 only showed aberrations proximal to the D11S925 locus at chromosome 11q, and the LOH in these tumours included the 11q22 region. Tumours 1071, 1201 and 1121 only had LOH at the 11p region.

*Southern analysis*

Southern analysis was carried out to distinguish between amplification and deletions at chromosome 11q. DNA from 17 of the 46 tumours that showed LOH at 11q with microsatellites was available for Southern hybridisation. We probed for four different loci: one at 11q13 (FGF3) and three at 11q22-qter (MMP3, D11S144 and D11S34). We were not able to detect a single amplification with the three probes that we used at 11q22–qter. However, Southern analyses proved that eight of the ten tumours, available for Southern analyses and showing LOH with the FGF3 microsatellite marker, were amplified. The amplification in each sample was estimated by titration to range from 2- to about 20-fold (data not shown). Figure 3 shows the pattern of LOH detected by PCR *vs* the results of the Southern analysis for the eight tumours having amplification at 11q13.3 and the two tumours that had no amplification. Tumours 1120, 795, 1110, 549, 975, 1216 and 1154 all exhibited the pattern of amplification at the 11q13 region and LOH at the more distal part of chromosome 11q. Tumour 981 only had amplification at 11q13 (i.e. no LOH) and tumours 982 and 799 had most likely lost the whole chromosome since all informative microsatellite markers showed LOH. Figure 4 shows the Southern results for two of the samples analysed.

### *Statistical analysis*

Table II shows the results from the chi-square analysis of association between LOH at chromosome 11 and clinicopathological factors. A statistically significant association was found between LOH at 11p (D11S922) and positive nodes, negative ER and PgR receptor status, high S-phase fraction, and non-diploidy. A significant association was found between LOH at 11p13 (D11S907) and positive node status and negative ER and PgR status. A significant association was found between LOH at 11q13 (FGF3 and D11S527) and positive node status and also between LOH at 11q24 (D11S969) and node positive tumours.

The statistical analysis showed a significant association between LOH at the 11q22–qter region and a high S-phase fraction. The only significant association observed between LOH and the family coefficient was at 11q13.5 (D11S527). No association was detected between LOH at any region of chromosome 11 and tumour size, tumour type or age of the patients at diagnosis. A significant association was found between LOH at 17q21 and LOH at 11p15. Two LOH regions at chromosome 11 (11p15 and 11q22–qter) showed significant association with LOH at chromosome 3p. No significant association was observed between LOH at chromosome 17p (p53) and chromosome 11 (Table III).

**Figure 2** Tumours that were informative for mapping of possible target regions of LOH at chromosome 11q. ☐, Heterozygote, ■, allelic imbalance; ▨, not informative.

**Figure 3** Results from Southern analysis of tumours that showed LOH for microsatellites. ■, allelic imbalance; ▤, amplification; ▨, not informative or not done; ☐, loss of heterozygosity; ☐, heterozygote; ▤ , homozygote, no amplification. Probes used in Southern analysis are shaded. *, Male breast cancer.

**Figure 4** Results from Southern analysis of two samples, both exhibiting LOH at 11q22 and amplification at 11q13. (**a**) Samples probed with SS6 (FGF3). (**b**) Samples probed with STMY1 (MMP3). (**c**) Samples probed with HM2A (c-MOS) for comparison of DNA content. N, normal DNA; T, tumour DNA.

Table II Results from statistical analysis of genetic alteration and clinicopathological factors

| Markers | ER status | PgR status | Tumour type | Node status | Tumour size | Age at diagnosis | S-phase fraction | Ploidy status | Family coefficient |
|---|---|---|---|---|---|---|---|---|---|
| D11S922 | 0.0001*** | 0.004** | NS | 0.012* | NS | NS | 0.0023** | 0.023* | NS |
| D11S907 | 0.019* | 0.0065** | NS | 0.016* | NS | NS | NS | NS | NS |
| D11S903 | NS | NS | NS | NS | NS | NS | NS | NS | NS |
| FGF3 | NS | NS | NS | 0.0092** | NS | NS | NS | 0.03* | NS |
| D11S527 | NS | NS | NS | 0.0053** | NS | NS | NS | NS | 0.025* |
| D11S35 | NS | NS | NS | NS | NS | NS | 0.0086** | NS | NS |
| D11S927 | NS | NS | NS | NS | NS | NS | 0.04* | NS | NS |
| D11S925 | NS | NS | NS | NS | NS | NS | 0.04* | NS | NS |
| D11S912 | NS | NS | NS | NS | NS | NS | 0.018* | NS | NS |
| D11S969 | NS | NS | NS | 0.03* | NS | NS | 0.0014** | NS | NS |

*95%, **99%, ***99.9%, NS, not significant. 11p15 (D11S922 and D11S907) was associated with negative oestrogen and progesterone receptor content, positive node status, high S-phase fraction and non-diploid status. 11q13 (FGF3 and D11S527) was associated with positive nodes and (for D11S527) having at least one first-degree or second-degree relative with breast cancer. 11q22-qter associated with high S-phase fraction and D11S969 with node positive breast cancer.

Table III Association of LOH at chromosome 11 with LOH at chromosome 17 and the 3p region

| | | 11p15 LOH | 11p15 ROH | | 11q13 LOH | 11q13 ROH | | 11q22-qter LOH | 11q22-qter ROH | |
|---|---|---|---|---|---|---|---|---|---|---|
| 17p13 | LOH | 8 | 20 | | 8 | 25 | | 11 | 22 | |
| (p53) | ROH | 5 | 38 | | 10 | 39 | | 17 | 34 | |
| | *P*-value | | | 0.07 | | | 0.68 | | | 1.00 |
| 17q21 | LOH | 13 | 10 | | 10 | 18 | | 12 | 16 | |
| (*BRCA1*) | ROH | 4 | 61 | | 15 | 63 | | 24 | 54 | |
| | *P*-value | | | 0.000*** | | | 0.08 | | | 0.25 |
| 3p | LOH | 11 | 13 | | 11 | 20 | | 15 | 14 | |
| region | ROH | 4 | 47 | | 13 | 43 | | 17 | 41 | |
| | *P*-value | | | 0.000*** | | | 0.22 | | | 0.04* |

The figures denote the number of informative samples that were analysed at the chromosomal regions. The chi-square test was used to determine significance. LOH, loss of heterozygosity; ROH, retention of heterozygosity.

### Discussion

It is well established that chromosome 11 is frequently altered in human breast cancer. Until now two regions, 11p15 and 11q13, have received most attention (Ali *et al.*, 1987; Varley *et al.*, 1988). The results presented here confirm that one additional region on chromosome 11 (11q22-qter) is altered in breast cancer (Carter *et al.*, 1994). The regions showing the highest frequency of LOH were 11p15, 11q22-q23.3 and 11q24-qter. In some tumours these were the only regions found to be altered (Figure 2). Frequent LOH telomeric to 11q23 has not been described previously in breast cancer. Whether the 11q22-q23.3 and 11q24-qter regions are both target regions for deletion or whether one is only a subregion of the other remains to be shown. A fine-scale microsatellite mapping in a larger number of tumour samples could provide information necessary to answer this question.

We could not detect amplification at the 11q22-qter region by Southern analysis. Therefore we conclude that the LOH detected by the microsatellite markers are deletions. This region is also frequently found to be deleted in ovarian cancer (Foulkes *et al.*, 1993).

Mapping of amplifications *vs* deletions demonstrates that the use of conventional PCR results alone to draw conclusions about deletion or amplification is questionable. Tumours 1110 and 549 are examples of cases where a clear-cut boundary between amplification and deletion cannot be determined from a map of LOH created by PCR analysis (Figure 3).

Our results from statistical analysis of clinicopathological variables and LOH at 11p15.5 and 11q13 support previously published results. A signficant association was found between LOH at 11p15.5 and 11p13-q13 (D11S922 and D11S907 respectively) and negative hormone receptor status and positive nodes. LOH at 11p15 (D11S922) was also associated with high S-phase fraction and non-diploid status. Similar results regarding nodes and hormone receptors have been reported by Ali *et al.* (1987) and Takita *et al.* (1992). We found no correlation with LOH at 11p and tumour size, age of onset or tumour type.

Previous studies have shown a significant association between amplification at 11q13 and positive nodes (Adnane *et al.*, 1989), positive oestrogen receptors and shorter life expectancy of those who were node negative (Borg *et al.*, 1991). The present results showed no association between positive hormone receptors and amplification at 11q13. However, our results showed association between LOH at 11q13 and node positive breast cancer. LOH detected with the D11S527 marker was also associated with having at least one first-degree or second-degree relative with breast cancer. Whether that has anything to do with the vicinity of the *MEN-1* region, remains to be shown.

In this study the highest frequency of LOH was detected at 11q22 (markers D11S35 and D11S927) and 11q24-qter (telomeric to the D11S925 marker). The same regions are thought to be involved in AT. The AT group A and C pedigrees show linkage to the 11q22-q23.1 region (Gatti *et al.*, 1988) and a candidate gene that corrects for the radiosensitivity in AT group D fibroblasts has been cloned from the 11q23.3-q24 region (Kapp *et al.*, 1992). Epidemiological studies have suggested that AT carriers are at a 5-fold risk of breast cancer (Swift *et al.*, 1991). Wooster *et al.* (1993) found no evidence of linkage to the AT region in familial breast cancer. We have found LOH at 11q22-qter in breast cancer cases from a family with a convincing linkage to *BRCA1* (J Gudmundsson *et al.*, unpublished results). Therefore the possible involvement of the AT region in the development of breast cancer in members of high risk breast cancer families is not ruled out. The molecular basis of AT is thought to be an abnormality of DNA repair (Hanawalt and Painter, 1985). The fact that LOH at 11q22-qter correlates with a high S-phase fraction raises the question whether the AT gene might be involved in the control of DNA synthesis. Determination of the frequency of LOH at chromosome 11q22-qter in AT carriers with breast cancer could be of help in clarifying that supposition.

The absence of association of LOH at 11q22-qter with the progesterone receptor, which has been mapped to the 11q22-q23 region (Rousseau-Merck *et al.*, 1987), seems to be comparable to the absence of correlation between oestrogen receptor and LOH at chromosome 6q (Magdelénat *et al.*, 1994). A possible explanation could be that having one copy of these hormone receptor genes is sufficient for the cells and also that a strong selection exists against mutations in these genes.

A significant correlation has been described by Takita *et al.* (1992) and Carter *et al.* (1994) between LOH at 17p13 and LOH at chromosome 11 (11p15 and 11q22-q23 respectively). Our results showed no significant association between LOH at chromosome 11 and 17p. On the other hand a highly significant association was found between LOH at 11p15 and LOH at 17q21 and 3p, which is interesting in our opinion in view of the location of the *BRCA1* gene at 17q21. Eiriksdottir *et al.* (1995) found LOH at chromosome 3p to be a significant prognostic variable for overall survival of breast cancer patients. The significant association between LOH at 11p15 and 3p could therefore have some prognostic value. Although only a few samples were examined, the incidence of LOH at chromosome 11q in male breast cancer is of interest. We think therefore that further investigations at the 11q region should be made with more samples of male breast cancer cases.

Chromosome 11 has been shown to suppress malignancy in cell hybrids. This supports the idea that chromosome 11 includes tumour suppressor gene(s). Introduction of the q-arm of chromosome 11 into HeLa cells was shown to suppress malignancy (Misra and Srivatsan, 1989). When a whole chromosome 11 was transferred into an MCF-7 breast cancer cell line, tumorigenicity was suppressed. Further refinement of tumour-suppressor gene(s) location implied the possibility of two genes, at 11p15.5 and 11q13-q23 (Negrini *et al.*, 1994). These conclusions were based upon the frequent findings of LOH on 11p15.5. Our results suggest that the 11q23-qter region may be just as likely to contain the suppressor of malignancy. Both of these regions (i.e. 11p15.5 and 11q23-qter) were deleted in a subclone of MCF-7 cells that still possessed malignancy (Negrini *et al.*, 1994). A tumour suppressor gene might also be localised to the common region (11q22-q23) of these two studies (Negrini *et al.*, 1994 and the present one) because the MCF-7 cells that retained this region had lower tumorigenicity. The possibility of two suppressor genes, located at the 11q22-q23.3 and q24-qter region, should therefore not be excluded.

**Acknowledgements**
The authors wish to thank Dr Å Borg, Dr NK Spurr and Dr R White for supplying the SS6, STMY1 and MCT128.1 Southern probes. The phi 2-11-2.2 probe was purchased from the ATCC. We also thank Professor Jonas Hallgrimsson for continuing support and his staff at the Department of Pathology for providing pathological material, the Icelandic Cancer Registry, and the Nordic Primer Bank of the Department of Clinical Genetics, University Hospital, Uppsala, Sweden for providing microsatellite markers. The Nordic Primer Bank is supported by the Nordic Council of Ministers. This work was supported by grants from the University of Iceland Graduate Research Fund, the Nordic Cancer Union, the Icelandic Cancer Society, the Memorial Fund of Bergthora Magnusdottir and Jakob B Bjarnason, the Science Fund of Iceland, and the Science Fund of the University Hospital of Iceland.

**References**

ADNANE J, GAUDREY P, SIMON M-P, SIMONY-LAFONTAINE J, JEANTEUR P AND THEILLET C. (1989). Proto-oncogene amplification and human breast tumour phenotype. *Oncogene*, **4**, 1389-1395.

ALI IU, LIDEREAU R, THEILLET C AND CALLAHAN R. (1987). Reduction to homozygosity of genes on chromosome 11 in human breast neoplasia. *Science*, **238**, 185-187.

BORG Å, SIGURDSSON H, CLARK GM, FERNÖ M, FUQUA SAW, OLSSON H, KILLANDER D AND MCGUIRE WL. (1991). Association of *INT2 HST1* coamplification in primary breast cancer with hormone-dependent phenotype and poor prognosis. *Br. J. Cancer*, **63**, 136-142.

BROWN DL, GAULT J, THOMPSON MB, HAUGE XY, EVANS GA AND LITT M. (1989). Dinucleotide repeat polymorphism at the D11S527 locus. *Nucleic Acids Res.*, **19**, 4790.

CARTER SL, NEGRINI M, BAFFA R, GILLUM DR, ROSENBERG AL, SCHWARTZ GF AND CROCE CM. (1994). Loss of heterozygosity at 11q22-q23 in breast cancer. *Cancer Res.*, **54**, 6270-6274.

CHEN LC, DOLLBAUM C AND SMITH H. (1989). Loss of heterozygosity on chromosome 1q in human breast cancer. *Proc. Natl Acad. Sci. USA*, **86**, 7204-7207.

CROPP CS, LIDEREAU R, CAMPBELL G, CHAMPEME MH AND CALLAHAN R. (1990). Loss of heterozygosity on chromosomes 17 and 18 in breast carcinoma: two additional regions identified. *Proc. Natl Acad. Sci. USA*, **87**, 7737-7741.

DEVILEE P, VAN VLIET M, VAN SLOUN P, DIJKSHOORN NK, HERMANS J, PEARSON PL AND CORNELISSE CJ. (1991). Allotype of human breast carcinoma: a second major site for loss of heterozygosity is on chromosome 6q. *Oncogene*, **6**, 1705-1711.

EIRIKSDOTTIR G, BERGTHORSSON JB, SIGURDSSON H, GUDMUNDSSON J, SKIRNISDOTTIR S, EGILSSON V, BARKARDOTTIR RB AND INGVARSSON S. (1995). Mapping of chromosome 3 alterations in human breast cancer using microsatellite PCR markers: correlation with clinical variables. *Int. J. Oncol.*, **6**, 369-375.

ESCOT C, THEILLET C, LIDEREAU R, SPYRATOS F, CHAMPEME M-H, GEST J AND CALLAHAN R. (1986). Genetic alterations of the c-*myc* oncogene (MYC) in human primary breast carcinomas. *Proc. Natl Acad. Sci. USA*, **83**, 4834-4838.

FERTI-PASSANTONOPOULOU A, PANANI AD AND RAPTIS S. (1991). Preferential involvement of 11q23-24 and 11p15 in breast cancer. *Cancer Genet. Cytogenet.*, **51**, 183-188.

FOULKES WD, CAMPBELL IG, STAMP GWH AND TROWSDALE J. (1993). Loss of heterozygosity and amplification on chromosome 11q in human ovarian cancer. *Br. J. Cancer*, **67**, 268-273.

GATTI RA, BERCEL I, BODER E, BRAEDT G, CHARMLEY P, CONCANNON P, ERSOY F, FOROUD T, JASPERS NG AND LANGE K. (1988). Localization of an ataxia telangiectasia gene to chromosome 11q22-23. *Nature*, **336**, 577-580.

GENUARDI M, TSIHIRA H, ANDERSON DE AND SAUNDERS GF. (1989). Distal deletion of chromosome 1p in ductal carcinoma of the breast. *Am. J. Hum. Genet.*, **45**, 73-82.

GRUIS NA, ABELN ECA, BARDOEL AFJ, DEVILEE P, FRANTS RR AND CORNELISSE CJ. (1993). PCR-based microsatellite polymorphisms in the detection of loss of heterozygosity in fresh and archival tumour tissue. *Br. J. Cancer*, **68**, 308-313.

GYAPAY G, MORISSETTE J, VIGNAL A, DIB C, FIZAMES C, MILLASSEAU P, MARC S, BERNARDI G, LATHROP M AND WEISSENBACH J. (1994). The 1993-94 Généthon human genetic linkage map. *Nat. Genet.*, **7**, 246-339.

HANAWALT P AND PAINTER R. (1985). On the nature of a DNA processing defect in ataxia-telangiectasia. In *Ataxia telangiectasia: Genetics, Neuropathology and Immunology of a Degenerative Disease of Childhood*. Gatti R and Swift M (eds) pp. 67-71. AR Liss: New York.

KALLIONIEMI A, KALLIONIEMI O-P, PIPER J, TANNER M, STOKKE T, CHEN L, SMITH HS, PINKEL D, GRAY JW AND WALDMAN FM. (1994). Detection and mapping of amplified DNA sequences in breast cancer by comparative genomic hybridization. *Proc. Natl Acad. Sci. USA*, **91**, 2156-2160.

KAPP LN, PAINTER RB, YU LC, VAN LOON N, RICHARD CW, JAMES MR, COX DR AND MURNANE JP. (1992). Cloning of a candidate gene for ataxia-telangiectasia group D. *Am. J. Hum. Genet.*, **51**, 45-54.

LARSSON C, SKOGSEID B, ÖBERG K, NAKAMURA Y AND NORDENSKJÖLD M. (1988). Multiple endocrine neoplasia type 1 gene maps to chromosome 11 and is lost in insulinoma. *Nature*, **332**, 85-87.

LINDBLOM A, SANDELIN K, ISELIUS L, DUMANSKI J, WHITE I, NORDENSKJÖLD M AND LARSSON C. (1994). Predisposition for breast cancer in carriers of constitutional translocation 11q;22q. *Am. J. Hum. Genet.*, **54**, 871–876.

LITT M, SHARMA V AND LUTY JA. (1990). Dinucleotide repeat polymorphism at the D11S35 locus. *Nucleic Acids Res.*, **18**, 5921.

LUNDBERG C, SKOOG L, CAVENEE WK AND NORDENSKJÖLD M. (1987). Loss of heterozygosity in human ductal breast tumours indicates a recessive mutation on chromosome 13. *Proc. Natl Acad. Sci. USA*, **84**, 2372–2376.

MACKAY J, STEEL CM, ELDER PA, FORREST APM AND EVANS HJ. (1988). Allele loss on short arm of chromosome 17 in breast cancer. *Lancet*, **2**, 1384–1385.

MADDEN SL, COOK DM, MORRIS JF, GASHLER A, SUKHATME VP, RAUSCHER III FJ. (1991). Transcriptional repression mediated by the WT1 Wilms tumour gene product. *Science*, **253**, 1550–1553.

MAGDELÉNAT H, GERBAULT-SEUREAU M AND DUTRILLAUX B. (1994). Relationship between loss of estrogen and progesterone receptor expression and of 6q and 11q chromosome arms in breast cancer. *Int. J. Cancer*, **57**, 63–66.

MILLER SA, DYKES DD AND POLESKY HF. (1988). A simple salting out procedure for extracting DNA from human nucleated cells. *Nucleic Acids Res.*, **16**, 1215.

MISRA BC AND SRIVATSAN ES. (1989). Localization of HeLa cell tumour-suppressor gene to the long arm of chromosome 11. *Am. J. Human Genet.*, **45**, 567–577.

NEGRINI M, SABBIONI S, POSSATI L, RATTAN S, CORALLINI A, BARBANTI-BRODANO G AND CROCE CM. (1994). Suppression of tumorigenicity of breast cancer cells by microcell-mediated chromosome transfer: studies on chromosomes 6 and 11. *Cancer Res.*, **54**, 1331–1336.

POLYMEROPOLUS MH, XIAO H, RATH DS AND MERRIL CR. (1990). Dinucleotide repeat polymorphism at the int-2 proto-oncogene locus (INT2). *Nucl. Acids Res.*, **18**, 7468.

ROUSSEAU-MERCK MF, BERNHEIM A, CHERIF D, MIGLIERINA R, MISRAHI M, LOOSFELT H, MILGRON E AND BERGER R. (1987). Localization of the human progesterone receptor gene (PGR) to chromosome 11q22–23. *Cytogenet. Cell Genet.*, **46**, 685.

SAMBROOK J, FRITSCH EF AND MANIATIS T. (1989). *Molecular cloning: A laboratory manual*, 2nd edn. Cold Spring Harbor Press: New York.

SATO T, TANIGAMI A, YAMAKAWA K, AKIYAMA F, KASUMI F, SAKAMOTO G AND NAKAMURA Y. (1991). Allelotype of breast cancer: Cumulative allele losses promote tumour progression in primary breast cancer. *Cancer Res.*, **50**, 7184–7189.

SWIFT M, MORRELL D, MASSEY RB AND CASE CL. (1991). Incidence of cancer in 161 families affected by ataxia telangiectasia. *New Eng. J. Med.*, **325**, 1831–1836.

TAKITA K, SATO T, MIYAGI M, WATATANI M, AKIYAMA F, SAKAMOTO G, KASUMI F, ABE R AND NAKAMURA Y. (1992). Correlation of loss of alleles on the short arms of chromosomes 11 and 17 with metastasis of primary breast cancer to lymph nodes. *Cancer Res.*, **52**, 3914–3917.

VARLEY JM, WALKER RA, CASEY G AND BRAMMER WJ. (1988). A common alteration to the *int-2* proto-oncogene in DNA from primary breast carcinomas. *Oncogene*, **3**, 87–91.

WOOSTER R, FORD D, MANGIAN J, PONDER BA, PETEO J, EASTON DF AND STRATTON MR. (1993). Absence of linkage to the ataxia telangiectasia locus in familial breast cancer. *Hum. Genet.*, **92**, 91–94.

YOKOTA J, TOYOSHIMA K, SUGIMURA T, YAMAMOTO T, TERADA M, BATTIFORA H AND CLINE MJ. (1986). Amplification of the *c-erbB-2* oncogene in human adenocarcinoma *in vivo*. *Lancet*, **2**, 765–766.